# Supplementary material for: Study protocol for a randomized controlled trial: Integrating the ‘Time-limited Trial’ in the emergency department
Source: PLoS One. 2024 Dec 23;19(12):e0313858. doi: 10.1371/journal.pone.0313858 (PMC11666031; doi:10.1371/journal.pone.0313858)
Supplement: S1 File — (DOCX) [file pone.0313858.s002.docx]

**Institutional Review Board**

**Intervention/Interaction Detailed Protocol**

Principal Investigator: Kei Ouchi, MD, MPH

Project Title: Time-limited Trials in the Emergency Department: A Randomized Trial

Version Date: 2023.08.16

Version Name/Number: Version 1

1. **BACKGROUND AND SIGNIFICANCE**

In the U.S., approximately 87 older adults (≥ 65 years) require intensive care in emergency departments (ED) every hour.^1^ This often occurs without patients understanding the high risk for newly acquired disability.^2^ During the last six months of life, 75% of older adults visit the ED,^3^ and intensive care is started in 30% of these patients.^4^ Intensive care use in this population increased from 18.5% in 1993 to 24.7% in 2002,^5^ resulting in an annual healthcare expenditure of $108 billion (2010, 4.1% of national healthcare expenditure).^6^ Shared decision-making plays a key role in this population, with more than 70% of older adults reporting that at the end of life, they prefer to focus on quality of life rather than life extension^7^ and would even give up one year of life to avoid dying in the intensive care unit (ICU).^8^ Despite a strong preference against invasive interventions, a systematic review revealed that 56% to 99% do not have advance directives at the time of an ED visit,^9^ reducing the likelihood of successful shared decision-making for initiation of intensive care. Since the decision to initiate intensive care occurs in the ED, emergency clinicians recognize the opportunity to provide patient-centered care.^10^ However, in the time-pressured ED environment, no standardized methods exist to guide emergency clinicians in leading critical shared decision-making regarding initiating intensive care.^11-13^

Experts agree that serious illness care should focus on preparing people for and then supporting them in in-the-moment decision-making during clinical deterioration.^14-16^ Medical crises exemplify the most challenging, in-the-moment decision-making. During medical crises, **time-limited trials (TLT)** are a recommended approach to reducing potentially nonbeneficial treatments for seriously ill older adults.^17-19^ TLT is a structured conversation between clinicians and patients/surrogates to discuss patients’ values and preferences, prognosis, and shared decision-making to use specific therapies for a prespecified period of time. TLT allows both clinicians and patients/surrogates to regularly assess if therapies are meeting patients’ goals over structured time intervals. Guided by the Capacity, Opportunity, Motivation to perform a Behavior (COM-B) framework,^20^ a quality improvement study of TLT in the ICU (N=209) was associated with a 35% increase in formal family meetings (p<0.01), 45% increase in clinicians eliciting values and preferences of patients (p<0.01), 1.3 day decrease in median ICU length of stay (p<0.02), and 13% decrease in mechanical ventilation (p=0.02). No change in hospital mortality was observed (58.4% vs. 58.3%, p=0.99).^21, 22^ TLTs have been published as an expert recommended approach in the official journal of the American College of Emergency Medicine.^23^ Although decisions to initiate intensive care occur in the ED, no study has evaluated the effect of TLT initiated in the ED.

The objective is to assess the feasibility and acceptability of TLT initiated in the ED. We will conduct a pilot randomized trial of TLT on seriously ill older adults starting intensive care in the ED. Rationale: Initiating TLT at the first moment of starting intensive care in the ED may further improve patient-centered outcome compare to the usual care (no TLT). Our central hypothesis is that TLT can be successfully initiated in a randomized study design in the ED.

**Impact:** We anticipate that introduction of TLT in the ED will lead to improved patient-centered utilization of intensive care for seriously ill older adult. If TLT is found to be feasible and acceptable in the ED, the potential exists to standardize the most challenging, in-the-moment decision-making for seriously ill older adults to improve their quality of end-of-life care nationally.

1. **SPECIFIC AIMS AND OBJECTIVES**

Aim 1: Determine the clinician-reported feasibility of conducting a clinician-level, randomized controlled trial of TLT compared to usual care in the ED.

Hypothesis: TLT is feasible to conduct a clinical-level randomized study in the ED.

Aim 2: Determine the patient-reported acceptability of TLT in the ED.

Hypothesis: The patients or surrogates (if patients are unable to consent) randomized to TLT will feel more heard and understood about their end-of-life care wishes (primary outcome).

1. **GENERAL DESCRIPTION OF THE STUDY DESIGN**

Overview

To demonstrate the feasibility (Aim 1) and acceptability (Aim 2) of TLT, we will conduct a parallel group, clinician-level, pilot randomized clinical trial among 40 emergency clinicians (~20 in TLT and ~20 in usual care) in the ED.

Randomization: Using REDCap’s integrated randomization module,^24^ we will conduct clinician-level randomization using random blocks of four to ensure that emergency clinicians are evenly distributed one-to-one between intervention and control arms.

**Aim 1: Feasibility**

We will conduct the randomized trial and measure the feasibility outcomes (e.g., time it takes to conduct the TLT conversations, see Outcomes below).

**Aim 2: Patient-Reported Acceptability**

Among the patients who were cared by the emergency clinicians enrolled in the randomized trial, we will measure the patient-reported acceptability of the TLT conversations (intervention arm only) and track their clinical outcomes via electronic health record (EHR) review (both intervention and control arms).

1. **SUBJECT SELECTION**

**Aim 1:** **Feasibility**

Emergency clinicians are subjects given they will answer few questions (i.e., clinician-reported feasibility questionnaire) after conducting the TLT conversations in the ED. Emergency clinicians will be randomized to intervention (TLT training) vs. usual care (no TLT training).

Inclusion: Attending physicians, resident physicians, or mid-level providers working in the ED who are willing to be randomized to become the study interventionists.

Exclusion: Emergency clinicians unwilling to consent and be randomized to intervention TLT training.

Recruitment: With the Chair’s approval and after reviewing the ED roster, the PI will announce the study in the staff meetings and solicit participation.

**Aim 2: Patient-reported Acceptability**

The subjects will be seriously ill older adults, or their surrogates being cared for by the participating emergency clinicians (both the intervention or control arms – see details on which outcome to be assessed for which arm in *6 Study Procedures, Outcome Assessment* below). If the emergency clinicians determine that the patient is not able to provide consent due to cognitive impairment, dementia, delirium, or critical illness, the surrogates will participate in the TLT conversations (Table 1).

Inclusion:

a. ≥50 years or older with ≥one serious life-limiting illness* being admitted to intensive care unit in the ED; or

b. ≥75 years or older being admitted to intensive care unit in the ED; or

c. ED clinicians will not be surprised if the patient died in the current hospital admission; and

d. English speaking

*Serious illness criteria with high one-year mortality were selected based on best practice recommendations^25^ similar to prior studies:^26-28^ 1) stage III/IV or metastatic cancer;^29, 30^ 2) end-stage renal disease on dialysis;^31^ 3) chronic heart/lung disease requiring home oxygen supplementation or experiencing shortness of breath with walking;^32, 33^ 4) moderate to severe dementia (surrogate required for enrollment); or 5) ≥2 hospitalizations in the past six months.^29, 34, 35^

Exclusion:

a. Unable or unwilling to provide informed consent; or

b. Non-English speaking; or

c. Clinically inappropriate, determined by emergency clinicians, and no surrogate is available

Recruitment:

| **Table 1. Intervention target and outcome assessment** | | |
| --- | --- | --- |
| **Ability to consent** | **Intervention target** | **Outcome assessment** |
| Present | Patient or surrogate (if patient unwilling due to critical illness) | Patient-reported acceptability + EHR |
| Absent | Surrogate | Surrogate-reported acceptability + EHR |

1. *How individuals are identified for recruitment including description of use of recruitment materials such as flyers, brochures, advertisements, letters, etc.*

🡪 In the ED, we will identify potential subjects by reviewing patients being cared for by the study interventionist clinicians from 8 AM to 10 PM, seven days per week using our EHR. No flyers, brochures, advertisements, or other promotional materials will be used.

🡪 Given the minimal risk of this study, our study team will provide a study information sheet for verbal consent.

1. *Who is responsible for identifying and recruiting individuals?*

🡪 Upon identification of potential subjects, our research team member (e.g., research assistant) will approach the interventionist emergency clinicians to see if his/her patient can be recruited for this study to confirm the inclusion/exclusion criteria.

1. *When and where individuals are recruited?*

🡪 Potential subjects will be recruited in the ED while receiving care.

🡪 After the intervention emergency clinician completes the TLT conversation, our research team member (e.g., research assistant) will approach the potential subjects or the surrogate for enrollment and completion of the patient-/surrogate-reported acceptability outcomes in the ED.

1. *How recruitment goals match the prevalence rates of the condition/disease being studied and the populations most impacted by the condition/disease being studied?*

🡪 We will recruit from all patients arriving in the ED, which will reflect the prevalence rates of seriously ill older adults in the ED.

1. *Methods to enhance enrollment of diverse individuals and under-represented populations*

🡪 We will cap enrollments of non-Hispanic Whites at 70% of the enrollment target and overenroll other under-represented minorities.

1. **SUBJECT ENROLLMENT**

**Aim 1: Feasibility**

After introducing the study at staff meetings, the study team will obtain verbal, informed consent with a study information sheet.

**Aim 2: Patient-reported Acceptability**

Pre-screening: We will identify potentially eligible patients using the ED track board in the EHR.

Consent Process:

1. Upon completion of TLT conversation by the interventionist emergency clinician (who also provides clinical care for the patient), our study team (e.g., research assistant, PI) will obtain verbal, informed consent from the patient or surrogate (if applicable) in the ED.
2. The capacity to consent the patient or surrogate is determined by the interventionist emergency clinician who conducted the TLT conversation.
3. **STUDY PROCEDURES**

**Intervention:** Clinician-led conversations about patient’s values and goals, potential benefits and risks of intensive care, and anticipated outcomes are standard of care in the ED. TLT is a multi-modal intervention that provides patient-centered structures to this standard of care. In this evaluation of TLT in the ED, the intervention components will include: 1) a structured conversation guide; 2) clinician training to use the guide; 3) electronic health records (EHR) template for documentation; and 4) standardized communication with the intensivist (**Fig 1**).

1. The TLT Conversation Guide: The structured conversation guide entails discussing patients’ values and goals, prognosis, and shared decision-making to use a trial of intensive care. Originally used in ICU settings,^21, 22^ we systematically refined the guide to be used in the ED incorporating inputs from patient advisors’ and emergency clinicians’ inputs (Appendix).
2. Clinician Training: Leveraging the study team’s expertise in serious illness communication training, the clinician training will include a one-hour didactic on research methodologies and serious illness communication skills, followed by a four-hour communication training with trained actors. Similar training has been described previously.^36-38^
3. EHR Documentation: An Epic template for documenting the TLT conversation findings has been developed (Appendix).
4. **
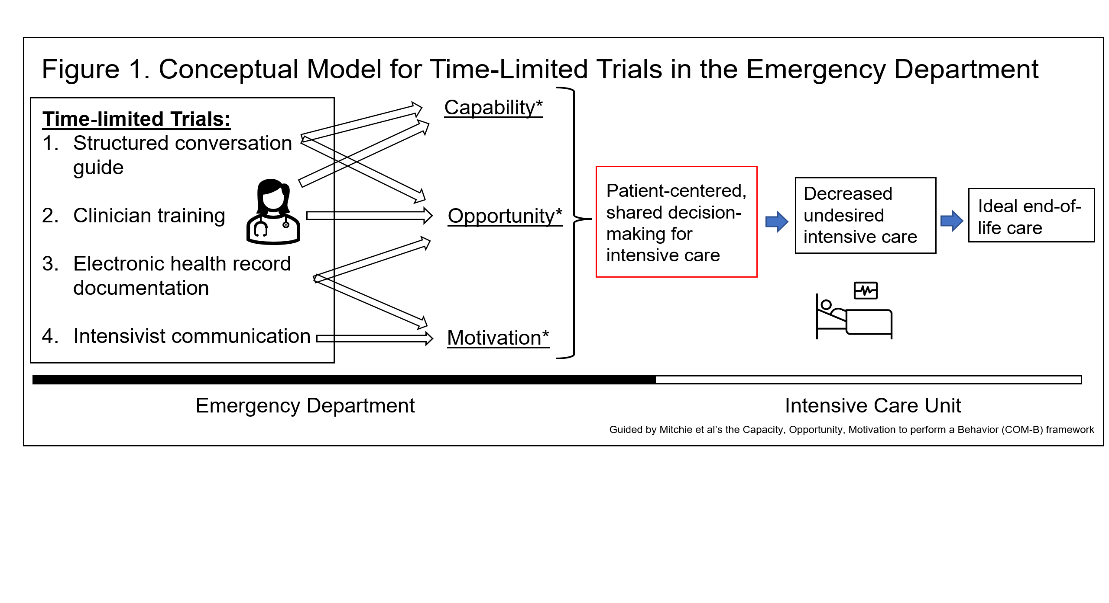
**Intensivist Communication: A standard template to communicate the TLT conversation findings to the intensivists has been developed (Appendix).

Control Group: Emergency clinicians assigned to the control group will not receive the TLT training/guide. Patients cared for by the control group emergency clinicians will receive usual care in the ED. No restrictions will be placed on serious illness conversations in the control group.

Outcome Assessments: We will collect the following variables during and after the TLT conversations (Table 2).

For Aim 1 Feasibility, we will collect all outcomes from the intervention arm, and EHR outcome from both intervention and control arms.

For Aim 2 Patient-Reported Acceptability, we will collect all outcomes from both the intervention and control arms.

*For clarity, we placed * on outcomes only collected from the intervention arm.*

| **Table 2. Outcomes and Assessment Timing** | | |
| --- | --- | --- |
| **Aim 1: Feasibility (* indicates outcomes collected only from the intervention arm)** | | |
| **Outcomes** | **Descriptions** | **Timing** |
| Time to complete the TLT conversation (primary outcome)* | With direct observation, the study team will record how long it takes to complete the TLT conversation by the intervenist ED clinician. | At the time of TLT conversation |
| Intervention fidelity* | With direct observation, the study team will record the completion of TLT components using an intervention fidelity checklist. | At the time of TLT conversation |
| Clinician-reported feasibility* | The study team will ask the interventionist emergency clinician to complete two 5-point Likert scale questions (i.e., “How feasible was it to conduct the TLT conversation with your patient?” and “How likely would you recommend the TLT conversation for your colleagues?” “Not at all (1)” to “Completely (5)”). | Immediately after the TLT conversation |
| Recruitment* | The percent that agree to conduct TLT conversations | At enrollment |
| Clinician-reported satisfaction* | The study team will ask the interventionist emergency clinician to complete 5-point Likert scale questions (i.e., “How satisfied are you with the conversation? “Not at all (1)” to “Completely (5)”). | Immediately after the TLT conversation |
| EHR documentation by inpatient clinicians (i.e., intensivists) | Review EHR for new documentation of reference to TLT, serious illness conversation, change in code status, or advance directive forms by inpatient clinicians. | After 24 hours, 48 hours, and 1 week |
| **Am 2: Patient-Reported Acceptability (* indicates outcomes collected only from the intervention arm)** | | |
| **Outcomes** | **Descriptions** | **Timing** |
| Patient-reported acceptability of TLT conversation (primary outcome)* | A 5-point Likert scale (i.e., “How acceptable was it for your doctor to talk to you about your expectations for ICU care?” and “How likely would you recommend this conversation for other patients like you?” “Not at all (1)” to “Completely (5)”). | Immediately after the TLT conversation |
| Heard and understood | A National Quality Forum endorsed, validated measure for palliative care modified to fit the context of serious illness conversations (“How well they feel heard and understood by your emergency department clinician about medical care they would want if they were to get sicker?” a 5-point Likert scale “Not at all (1)” to “Completely (5)”).^39^ | Immediately after the TLT conversation |
| Patient-reported end-of-life quality of communication | A validated, quality of end-of-life communication survey (4 items selected a priori, a 10-point Likert scale “Worst you can imagine (0)” to “Best you can imagine (10)”).^40^ | Immediately after the TLT conversation |
| Decisional regret scale | A validated survey that measures “distress or remorse after a [health care] decision” (5 items, 5-point Likert scale “Strongly agree (1)” to “Strongly disagree (5)”).^41^ | After 24 hours, 48 hours, and 1 week |
| Clinical outcomes abstracted from EHR | Number of days to the first family meeting in intensive care unit (ICU), ICU length of stay (LOS), hospital LOS, # family meetings, ICU procedures (e.g., CPR, pressors, etc.), ICU mortality, hospital disposition, hospice utilization. | After discharge or upon patient death |

A research assistant blinded to treatment allocation will perform all follow-up assessments. Our team will extract data from the EHR using a standardized approach.^42^

Study Termination:

We will follow the participating clinicians for up to one year to accrue an adequate number of patients they admit to the intensive care unit. Given that an emergency clinician may encounter ~3 to 10 seriously ill older adults being admitted to the intensive care unit per month, we estimate that it would take approximately one year to accrue ~10 patient/interventionist ED clinician who might meet the inclusion/exclusion criteria for Aim 2. The follow-up assessments of the patients will terminate when/if the participants die or are discharged from the hospital.

Remuneration: For Aim 1 Feasibility, we will compensate $500 to clinicians randomized to the intervention arm for one-hour didactic and four-hour communication training. In addition, we will compensate $25 for every patient that the TLT conversation is completed. For control arm clinicians, we will compensate $100 after randomization. For Aim 2 Patient-reported Acceptability, we will compensate $20 for completing the surveys.

Data Sharing: We do not anticipate sharing the data with collaborators outside Mass General Brigham.

1. **RISKS AND DISCOMFORTS**

Compared to the usual clinical care, we do not anticipate any additional risks or discomforts to our clinician or patient participants other than privacy/confidentiality risks. Several steps have been taken to maximize privacy. Questionnaires will be completed in private, walled ED treatment areas. Subjects will be assured that refusal to participate will not affect their care in any way. Unique identification numbers will be assigned to participants to link contact information and study data. These identification numbers do not contain patient identifying information. All research personnel involved in any way in this project will complete training in the protection of human research subjects per guidelines issued by the U.S. Department of Health and Human Services, Office for Human Research Protection. Staff training conducted by the candidate will include information about the importance of confidentiality and techniques to maintain confidentiality of all information reported by research participants. All study data will be collected and stored using REDCap. The purpose is to provide a system and associated support resources to enable efficient and high-quality collection and management of research data that is standards-based in design, development, and implementation. Standard features of electronic clinical research data management systems are available in the web-based systems provided with the service. These include interactive data entry with real-time field validation, lab data imports, audit logs to record database modifications, database integrity checks, security (in logins, permissions based on need, and encryption), reporting, forms inventory, and exports to common statistical packages for analysis. The database system provides for secure web-based data entry with the data stored on servers that maintained by Mass General Brigham. The data is encrypted during transmission. Consistent with NIH guidelines, a Data Safety Monitoring Committee (DSMC) will not be required for this single site, interventional trial.

1. **BENEFITS**

No proven benefits to the participants are expected. However, we hope that providing patient-centered structures to the usual care will improve the alignment of intensive care utilization with patients’ values and goals.

1. **STATISTICAL ANALYSIS**

**Aim 1: Determine the feasibility of conducting a clinician-level, randomized controlled trial of TLT compared to usual care.**

Hypothesis: TLT is feasible to conduct a clinical-level randomized study in the ED.

Descriptive statistics will be used for baseline characteristics of the study sample. We will consider the feasibility threshold to be the following:

- The median time to complete TLT conversations is <10 minutes (primary outcome).

- >70% of intervention components are completed, assessed using intervention fidelity checklist by an observer/research team member.

- >70% of clinicians in the intervention group reporting “somewhat feasible (4)” or “completely feasible (5).

We will track recruitment, participation, and retention rates of interventionist clinicians, including reasons for not participating. We will track which inpatient clinicians authored the follow-up serious illness conversations in EHR. The EHR-based outcome will be assessed using a EHR review. We will publish our findings regardless of the results and will follow the CONSORT guidelines for reporting RCT.^43^

**Aim 2: Determine the patient-reported acceptability of TLT in the ED.**

Hypothesis: The patients and surrogates undergoing TLT conversations will feel heard and understood about their end-of-life care wishes (primary acceptability outcome).

Descriptive statistics will be used for baseline characteristics of the study sample. We will compare patient demographics between study arms to assess randomization using two sample t-test or Wilcoxon rank sum test for continuous variables and chi-square for categorical variables. Within arms, we will use a one-sample binomial exact test of proportions for categorical outcomes (e.g., EHR documentation), and Wilcoxon signed ranks test for ordinal outcomes (e.g., Heard and Understood survey). A significance level, alpha, is set to 0.05. We will conduct a secondary analysis using linear mixed models at baseline and one month to include all non-missing data. The EHR-based outcome will be assessed using an EHR review. We will publish our findings regardless of the results and will follow the CONSORT guidelines for reporting RCT.^43^

**Sample Size:** No previous two-arm trial of TLT has been conducted; thus, the effect size remains unknown. Given the primary purpose of conducting a RCT is to demonstrate the feasibility of conducting a randomized design study, no gold standard for sample size calculations exists. However, prior literature suggest that 15 to 20 participants per group are required to ensure scientific validity of the pilot study results.^44^ Therefore, the present study will include a total of 32 participants with 16 participants per group. In addition, the results of this pilot study will provide information on the difference between groups and standard deviation necessary for sample size calculation during the future full-scale study.

1. **MONITORING AND QUALITY ASSURANCE**

**Describe the plans that will be followed by study staff for monitoring and quality assurance, including:**

- **Adverse event (AE) criteria and reporting procedures**

Any AE will be reported to our institutional review board at the time of annual continuing review by the principal investigator using an AE Form in accordance with the Partners Human Research Committee guidelines for AE reporting.

- **Planned safety monitoring**

The proposed research is a minimal risk study and does not meet the criteria for an NIH-defined Phase III trial given no patient outcomes will be collected. Therefore, a data and safety monitoring board will not be required, and the PI will be solely responsible for data and safety monitoring of the proposed research. In the event of an adverse event, the research team will report immediately to the PI, who will be responsible for management and reporting to the IRB, and to the NIH institute.

- **Outcomes monitoring, including planned frequency of review**

Not applicable.

- **Study stopping rules as applicable**

Unanticipated Problems (UP): As with any AE above, the PI will report any UP within 5 working days / 7 calendar days of the date. The investigator first becomes aware of the problem using an Adverse Event Form in accordance with the Partners Human Research Committee guidelines for AE reporting, please see attached institutional policy.

- **Internal monitoring of source data, protocol adherence, and recordkeeping, including which staff will be responsible and planned frequency of review**

The PI is responsible for monitoring and recordkeeping on REDCap.

- **Independent monitoring of source data as applicable**

All personal health information will be removed from the research data from the participants and clinicians at the earliest possible time. All data will be stored in a secured Mass General Brigham approved shared drive accessible only to the study staff. Recordings will be stored on an encrypted recording device and saved to the secure Mass General Brigham-approved shared drive.

1. **PRIVACY AND CONFIDENTIALITY**

Study procedures will be conducted in a private setting

Only data and/or specimens necessary for the conduct of the study will be collected

Data collected (paper and/or electronic) will be maintained in a secure location with appropriate protections such as password protection, encryption, physical security measures (locked files/areas)

Specimens collected will be maintained in a secure location with appropriate protections (e.g. locked storage spaces, laboratory areas)

Data and specimens will only be shared with individuals who are members of the IRB-approved research team or approved for sharing as described in this IRB protocol

Data and/or specimens requiring transportation from one location or electronic space to another will be transported only in a secure manner (e.g. encrypted files, password protection, using chain-of-custody procedures, etc.)

All electronic communication with participants will comply with Mass General Brigham secure communication policies

Identifiers will be coded or removed as soon as feasible and access to files linking identifiers with coded data or specimens will be limited to the minimal necessary members of the research team required to conduct the research

All staff are trained on and will follow the Mass General Brigham policies and procedures for maintaining appropriate confidentiality of research data and specimens

The PI will ensure that all staff implement and follow any Research Information Service Office (RISO) requirements for this research

Additional privacy and/or confidentiality protections

1. **REFERENCES**

1. Sjoding MW, Prescott HC, Wunsch H, Iwashyna TJ, Cooke CR. Longitudinal Changes in ICU Admissions Among Elderly Patients in the United States. Crit Care Med. 2016;44(7):1353-60. doi: 10.1097/CCM.0000000000001664. PubMed PMID: 26968023; PMCID: PMC4911310.

2. Riegel B, Huang L, Mikkelsen ME, Kutney-Lee A, Hanlon AL, Murtaugh CM, Bowles KH. Early Post-Intensive Care Syndrome among Older Adult Sepsis Survivors Receiving Home Care. J Am Geriatr Soc. 2019;67(3):520-6. Epub 20181130. doi: 10.1111/jgs.15691. PubMed PMID: 30500988; PMCID: PMC6402981.

3. Smith AK, McCarthy E, Weber E, Cenzer IS, Boscardin J, Fisher J, Covinsky K. Half of older Americans seen in emergency department in last month of life; most admitted to hospital, and many die there. Health Aff (Millwood). 2012;31(6):1277-85. doi: 10.1377/hlthaff.2011.0922. PubMed PMID: 22665840; PMCID: PMC3736978.

4. Yu W, Ash AS, Levinsky NG, Moskowitz MA. Intensive care unit use and mortality in the elderly. J Gen Intern Med. 2000;15(2):97-102. doi: 10.1046/j.1525-1497.2000.02349.x. PubMed PMID: 10672112; PMCID: PMC1495347.

5. Sharma G, Freeman J, Zhang D, Goodwin JS. Trends in end-of-life ICU use among older adults with advanced lung cancer. Chest. 2008;133(1):72-8. Epub 20071107. doi: 10.1378/chest.07-1007. PubMed PMID: 17989164; PMCID: PMC4034445.

6. Halpern NA, Pastores SM. Critical Care Medicine Beds, Use, Occupancy, and Costs in the United States: A Methodological Review. Crit Care Med. 2015;43(11):2452-9. doi: 10.1097/CCM.0000000000001227. PubMed PMID: 26308432; PMCID: PMC5520980.

7. Steinhauser KE, Christakis NA, Clipp EC, McNeilly M, McIntyre L, Tulsky JA. Factors considered important at the end of life by patients, family, physicians, and other care providers. JAMA. 2000;284(19):2476-82. PubMed PMID: 11074777.

8. Rubin EB, Buehler A, Halpern SD. Seriously Ill Patients' Willingness to Trade Survival Time to Avoid High Treatment Intensity at the End of Life. JAMA Intern Med. 2020;180(6):907-9. doi: 10.1001/jamainternmed.2020.0681. PubMed PMID: 32250436; PMCID: PMC7136854.

9. Oulton J, Rhodes SM, Howe C, Fain MJ, Mohler MJ. Advance directives for older adults in the emergency department: a systematic review. J Palliat Med. 2015;18(6):500-5. doi: 10.1089/jpm.2014.0368. PubMed PMID: 25763860.

10. Stone SC, Mohanty S, Grudzen CR, Shoenberger J, Asch S, Kubricek K, Lorenz KA. Emergency medicine physicians' perspectives of providing palliative care in an emergency department. J Palliat Med. 2011;14(12):1333-8. doi: 10.1089/jpm.2011.0106. PubMed PMID: 22136262.

11. Smith AK, Fisher J, Schonberg MA, Pallin DJ, Block SD, Forrow L, Phillips RS, McCarthy EP. Am I doing the right thing? Provider perspectives on improving palliative care in the emergency department. Ann Emerg Med. 2009;54(1):86-93, e1. doi: 10.1016/j.annemergmed.2008.08.022. PubMed PMID: 18930337.

12. Dy SM, Herr K, Bernacki RE, Kamal AH, Walling AM, Ersek M, Norton SA. Methodological Research Priorities in Palliative Care and Hospice Quality Measurement. J Pain Symptom Manage. 2016;51(2):155-62. doi: 10.1016/j.jpainsymman.2015.10.019. PubMed PMID: 26596877.

13. Kelley AS, Bollens-Lund E. Identifying the Population with Serious Illness: The "Denominator" Challenge. J Palliat Med. 2018;21(S2):S7-S16. doi: 10.1089/jpm.2017.0548. PubMed PMID: 29125784; PMCID: PMC5756466.

14. Curtis JR, Kross EK, Stapleton RD. The Importance of Addressing Advance Care Planning and Decisions About Do-Not-Resuscitate Orders During Novel Coronavirus 2019 (COVID-19). JAMA. 2020;323(18):1771-2. doi: 10.1001/jama.2020.4894. PubMed PMID: 32219360.

15. Curtis JR. Three Stories About the Value of Advance Care Planning. JAMA. 2021;326(21):2133-4. doi: 10.1001/jama.2021.21075. PubMed PMID: 34874415.

16. Morrison RS, Meier DE, Arnold RM. What's Wrong With Advance Care Planning? JAMA. 2021;326(16):1575-6. doi: 10.1001/jama.2021.16430. PubMed PMID: 34623373.

17. Quill TE, Holloway R. Time-limited trials near the end of life. JAMA. 2011;306(13):1483-4. doi: 10.1001/jama.2011.1413. PubMed PMID: 21972312.

18. Kon AA, Davidson JE, Morrison W, Danis M, White DB. Shared Decision-Making in Intensive Care Units. Executive Summary of the American College of Critical Care Medicine and American Thoracic Society Policy Statement. Am J Respir Crit Care Med. 2016;193(12):1334-6. doi: 10.1164/rccm.201602-0269ED. PubMed PMID: 27097019; PMCID: PMC4910896.

19. Curtis JR, Engelberg RA, Bensink ME, Ramsey SD. End-of-life care in the intensive care unit: can we simultaneously increase quality and reduce costs? Am J Respir Crit Care Med. 2012;186(7):587-92. Epub 20120802. doi: 10.1164/rccm.201206-1020CP. PubMed PMID: 22859524; PMCID: PMC3480521.

20. Michie S, van Stralen MM, West R. The behaviour change wheel: a new method for characterising and designing behaviour change interventions. Implement Sci. 2011;6:42. Epub 20110423. doi: 10.1186/1748-5908-6-42. PubMed PMID: 21513547; PMCID: PMC3096582.

21. Chang D, Parrish J, Kamangar N, Liebler J, Lee M, Neville T. Time-Limited Trials Among Critically Ill Patients With Advanced Medical Illnesses to Reduce Nonbeneficial Intensive Care Unit Treatments: Protocol for a Multicenter Quality Improvement Study. JMIR Res Protoc. 2019;8(11):e16301. Epub 20191125. doi: 10.2196/16301. PubMed PMID: 31763988; PMCID: PMC6902129.

22. Chang DW, Neville TH, Parrish J, Ewing L, Rico C, Jara L, Sim D, Tseng CH, van Zyl C, Storms AD, Kamangar N, Liebler JM, Lee MM, Yee HF, Jr. Evaluation of Time-Limited Trials Among Critically Ill Patients With Advanced Medical Illnesses and Reduction of Nonbeneficial ICU Treatments. JAMA Intern Med. 2021;181(6):786-94. doi: 10.1001/jamainternmed.2021.1000. PubMed PMID: 33843946; PMCID: PMC8042568.

23. Ouchi K, Lawton AJ, Bowman J, Bernacki R, George N. Managing Code Status Conversations for Seriously Ill Older Adults in Respiratory Failure. Ann Emerg Med. 2020;76(6):751-6. doi: 10.1016/j.annemergmed.2020.05.039. PubMed PMID: 32747084.

24. Harris PA, Taylor R, Thielke R, Payne J, Gonzalez N, Conde JG. Research electronic data capture (REDCap)--a metadata-driven methodology and workflow process for providing translational research informatics support. J Biomed Inform. 2009;42(2):377-81. doi: 10.1016/j.jbi.2008.08.010. PubMed PMID: 18929686; PMCID: PMC2700030.

25. Bernacki RE, Block SD, American College of Physicians High Value Care Task F. Communication about serious illness care goals: a review and synthesis of best practices. JAMA Intern Med. 2014;174(12):1994-2003. doi: 10.1001/jamainternmed.2014.5271. PubMed PMID: 25330167.

26. Grudzen CR, Shim DJ, Schmucker AM, Cho J, Goldfeld KS, Investigators EM. Emergency Medicine Palliative Care Access (EMPallA): protocol for a multicentre randomised controlled trial comparing the effectiveness of specialty outpatient versus nurse-led telephonic palliative care of older adults with advanced illness. BMJ Open. 2019;9(1):e025692. doi: 10.1136/bmjopen-2018-025692. PubMed PMID: 30813112; PMCID: PMC6347856.

27. Grudzen CR, Richardson LD, Johnson PN, Hu M, Wang B, Ortiz JM, Kistler EA, Chen A, Morrison RS. Emergency Department-Initiated Palliative Care in Advanced Cancer: A Randomized Clinical Trial. JAMA Oncol. 2016. doi: 10.1001/jamaoncol.2015.5252. PubMed PMID: 26768772.

28. Grudzen CR, Brody AA, Chung FR, Cuthel AM, Mann D, McQuilkin JA, Rubin AL, Swartz J, Tan A, Goldfeld KS, Investigators P-E. Primary Palliative Care for Emergency Medicine (PRIM-ER): Protocol for a Pragmatic, Cluster-Randomised, Stepped Wedge Design to Test the Effectiveness of Primary Palliative Care Education, Training and Technical Support for Emergency Medicine. BMJ Open. 2019;9(7):e030099. doi: 10.1136/bmjopen-2019-030099. PubMed PMID: 31352424; PMCID: PMC6661655.

29. Walter LC, Brand RJ, Counsell SR, Palmer RM, Landefeld CS, Fortinsky RH, Covinsky KE. Development and validation of a prognostic index for 1-year mortality in older adults after hospitalization. Jama. 2001;285(23):2987-94.

30. Levine SK, Sachs GA, Jin L, Meltzer D. A prognostic model for 1-year mortality in older adults after hospital discharge. The American Journal of Medicine. 2007;120(5):455-60.

31. Kurella Tamura M. Incidence, management, and outcomes of end-stage renal disease in the elderly. Curr Opin Nephrol Hypertens. 2009;18(3):252-7. doi: 10.1097/mnh.0b013e328326f3ac. PubMed PMID: 19374012; PMCID: PMC2738843.

32. Hardy SE, Kang Y, Studenski SA, Degenholtz HB. Ability to walk 1/4 mile predicts subsequent disability, mortality, and health care costs. J Gen Intern Med. 2011;26(2):130-5. doi: 10.1007/s11606-010-1543-2. PubMed PMID: 20972641; PMCID: PMC3019329.

33. Rolland Y, Lauwers-Cances V, Cesari M, Vellas B, Pahor M, Grandjean H. Physical performance measures as predictors of mortality in a cohort of community-dwelling older French women. Eur J Epidemiol. 2006;21(2):113-22. doi: 10.1007/s10654-005-5458-x. PubMed PMID: 16518679.

34. Creditor MC. Hazards of hospitalization of the elderly. Annals of Internal Medicine. 1993;118(3):219-23.

35. Fischer SM, Gozansky WS, Sauaia A, Min S-J, Kutner JS, Kramer A. A practical tool to identify patients who may benefit from a palliative approach: the CARING criteria. Journal of pain and symptom management. 2006;31(4):285-92.

36. Ouchi K, George N, Revette AC, Hasdianda MA, Fellion L, Reust A, Powell LH, Sudore R, Schuur JD, Schonberg MA, Bernstein E, Tulsky JA, Block SD. Empower Seriously Ill Older Adults to Formulate Their Goals for Medical Care in the Emergency Department. J Palliat Med. 2019;22(3):267-73. doi: 10.1089/jpm.2018.0360. PubMed PMID: 30418094; PMCID: PMC6391608.

37. Pajka SE, Hasdianda MA, George N, Sudore R, Schonberg MA, Bernstein E, Tulsky JA, Block SD, Ouchi K. Feasibility of a Brief Intervention to Facilitate Advance Care Planning Conversations for Patients with Life-Limiting Illness in the Emergency Department. J Palliat Med. 2020. doi: 10.1089/jpm.2020.0067. PubMed PMID: 32471321.

38. Leiter RE, Yusufov M, Hasdianda MA, Fellion LA, Reust AC, Block SD, Tulsky JA, Ouchi K. Fidelity and Feasibility of a Brief Emergency Department Intervention to Empower Adults With Serious Illness to Initiate Advance Care Planning Conversations. J Pain Symptom Manage. 2018;56(6):878-85. doi: 10.1016/j.jpainsymman.2018.09.003. PubMed PMID: 30223014; PMCID: PMC6289886.

39. Gramling R, Stanek S, Ladwig S, Gajary-Coots E, Cimino J, Anderson W, Norton SA, Group ARCW, Aslakson RA, Ast K, Elk R, Garner KK, Gramling R, Grudzen C, Kamal AH, Lamba S, LeBlanc TW, Rhodes RL, Roeland E, Schulman-Green D, Unroe KT. Feeling Heard and Understood: A Patient-Reported Quality Measure for the Inpatient Palliative Care Setting. J Pain Symptom Manage. 2016;51(2):150-4. doi: 10.1016/j.jpainsymman.2015.10.018. PubMed PMID: 26596879.

40. Engelberg R, Downey L, Curtis JR. Psychometric characteristics of a quality of communication questionnaire assessing communication about end-of-life care. J Palliat Med. 2006;9(5):1086-98. doi: 10.1089/jpm.2006.9.1086. PubMed PMID: 17040146.

41. Brehaut JC, O'Connor AM, Wood TJ, Hack TF, Siminoff L, Gordon E, Feldman-Stewart D. Validation of a decision regret scale. Med Decis Making. 2003;23(4):281-92. doi: 10.1177/0272989X03256005. PubMed PMID: 12926578.

42. Gilbert EH, Lowenstein SR, Koziol-McLain J, Barta DC, Steiner J. Chart reviews in emergency medicine research: Where are the methods? Ann Emerg Med. 1996;27(3):305-8. PubMed PMID: 8599488.

43. Moher D, Hopewell S, Schulz KF, Montori V, Gotzsche PC, Devereaux PJ, Elbourne D, Egger M, Altman DG. CONSORT 2010 explanation and elaboration: updated guidelines for reporting parallel group randomised trials. Bmj. 2010;340:c869. doi: 10.1136/bmj.c869. PubMed PMID: 20332511; PMCID: 2844943.

44. Hertzog MA. Considerations in determining sample size for pilot studies. Res Nurs Health. 2008;31(2):180-91. doi: 10.1002/nur.20247. PubMed PMID: 18183564.
