# Supplementary material for: Study protocol for a randomized controlled trial: Integrating the ‘Time-limited Trial’ in the emergency department
Source: PLoS One. 2024 Dec 23;19(12):e0313858. doi: 10.1371/journal.pone.0313858 (PMC11666031; doi:10.1371/journal.pone.0313858)
Supplement: S1 Appendix — (DOCX) [file pone.0313858.s003.docx]

**Time-Limited Trial Conversation Guide**

Version 2023.10.13

**I. UNDERSTANDING and ACUTE CARE NEEDS**

**1. Understanding of Condition**

*e.g., “Tell me what you understand of [patient]’s condition and what’s happened after arriving in the ED?”*

**2. Summarize the ED Course**

*e.g., "From our view, I want to make sure you know [XYZ] has happened, and we’re giving [xyz] treatment.”*

**3. Hope & Worry Statement**

*e.g., “Based on what we know so far, our hope is that [patient] improves with these treatments. At the same time, I also worry the treatments may not reverse the problem.”*

**4. Share Prognosis**

*e.g., "For many patients like [patient], ICU treatments are incredibly stressful (“put the body through a lot”), and often end with partial recovery. This might mean not returning home and having difficulty caring for [patient]. Do you have any questions so far?”*

**II. VALUES and PREFERENCES**

**1. Baseline Function and Perceived Quality of Life**

*e.g., “Before we talk further, I would like to know more about [patient]. Please help me understand what a ‘good day’ looked like before today.” (If not specific, ask probing questions).*

*e.g., “How would [patient] describe that life?” (If not specific, ask probing questions).*

**2. Would Not Want**

*e.g., “Has [patient] ever talked about medical care [patient] would not want? For example, breathing machines or dialysis.”*

**3. Important to the Patient**

*e.g., “What do you think [patient] would say is most important to [patient] at this time, many people would say spending more time at home, intensive treatments for diseases, staying as comfortable as possible, etc.?"*

**III. SUMMARY**

*e.g., “What I have heard is that [describe patient’s baseline function and perceived quality of life]. [XYZ] is important to [patient], and [xyz] would not be something [patient] is OK with – did I get that right? Is there anything that I might have missed that is important to [patient]?”*

**IV: TIME-LIMITED TRIAL**

**1. Treatment**

*e.g., “Based on what we’ve discussed, I think it makes sense to do a care plan of [treatment] for a limited time. Long enough to see if the treatment is working. Does this sound OK?”*

**2. ICU Expectations**

*e.g., “I am going to share what we talked about with the ICU team (and your disease specialist, if applicable). They will be able to tell you if the treatment is helping [patient] or not. Generally, this becomes clear in a few days or sooner depending on how the current treatments are working.”*

**3. Next Steps**

*e.g., “We will listen to what your body is telling us to see if the current care plan makes sense. If it is not helping, we would worry that we are causing suffering without benefit. In situations like that, many patients and families choose to stop unhelpful treatment and change care options.”*

**Section V: CONCLUSION**

**1. Clarify & Confirm**

*e.g., “To ensure we are on the same page, can you tell us how this plan feels to you?”*

**2. Thank you**

*e.g., “Thank you for your time. We will make sure the ICU team is aware of what we discussed.”*
