# Supplementary material for: Study protocol for a randomized controlled trial: Integrating the ‘Time-limited Trial’ in the emergency department
Source: PLoS One. 2024 Dec 23;19(12):e0313858. doi: 10.1371/journal.pone.0313858 (PMC11666031; doi:10.1371/journal.pone.0313858)
Supplement: Attachment — Submitted filename: Response Letter 2024.10.15ver2.docx [file pone.0313858.s005.docx]

Dr. Dong Wook Chang

Academic Editor

*PLOS ONE*

Re: *PLOS ONE* Decision: Revision required [PONE-D-24-11345] - [EMID:41bc29f2066e9e1a]

Dear Editors and Reviewers,

We thank the Editors and the Reviewers for their insightful and thoughtful comments. Please see the point-by-point responses to the Editors’ and Reviewers’ critiques below. Our responses are in blue.

**Editors:**

1. Please ensure that your manuscript meets PLOS ONE's style requirements, including those for file naming.

Thank you for pointing this out. We have revised to the PLOS One’s style requirement.

2. Thank you for stating the following in the Acknowledgments Section of your manuscript:

“Dr. Ouchi is supported by the National Institute on Aging (K76AG064434)” We note that you have provided funding information that is not currently declared in your Funding Statement. However, funding information should not appear in the Acknowledgments section or other areas of your manuscript. We will only publish funding information present in the Funding Statement section of the online submission form. Please remove any funding-related text from the manuscript and let us know how you would like to update your Funding Statement. Currently, your Funding Statement reads as follows:

“The author(s) received no specific funding for this work.” Please include your amended statements within your cover letter; we will change the online submission form on your behalf.

As mentioned in our previous correspondence, we have clarified that Dr. Ouchi’s funding has no relation to the current work being submitted. Therefore, we have removed the Funding Statement, ”Dr. Ouchi is supported by the National Institute on Aging (K76AG064434)” from our manuscript as instructed. The online submission form remains unchanged, with the statement: "The author(s) received no specific funding for this work."

3. Thank you for stating the following in the Competing Interests

“Dr. Ouchi has received funding personally from Jolly Good, Inc (a virtual reality company) for consulting.”We note that one or more of the authors are employed by a commercial company: Jolly Good, Inc.

a. Please provide an amended Funding Statement declaring this commercial affiliation, as well as a statement regarding the Role of Funders in your study.

b. Please also provide an updated Competing Interests Statement declaring this commercial affiliation along with any other relevant declarations relating to employment, consultancy, patents, products in development, or marketed products, etc.

Please include both an updated Funding Statement and Competing Interests Statement in your cover letter. We will change the online submission form on your behalf.

As mentioned in our previous correspondence, we have clarified that Jolly Good, Inc. has no relation to the current work being submitted. Therefore, we have removed the Competing Interests as well.

4. When completing the data availability statement of the submission form, you indicated that you will make your data available on acceptance. We strongly recommend all authors decide on a data sharing plan before acceptance, as the process can be lengthy and hold up publication timelines. Please note that, though access restrictions are acceptable now, your entire data will need to be made freely accessible if your manuscript is accepted for publication. This policy applies to all data except where public deposition would breach compliance with the protocol approved by your research ethics board. If you are unable to adhere to our open data policy, please kindly revise your statement to explain your reasoning and we will seek the editor's input on an exemption. Please be assured that, once you have provided your new statement, the assessment of your exemption will not hold up the peer review process.

Thank you for your comments. The current submission is a protocol manuscript without any pilot data. Therefore, no data will be available upon its acceptance or publication of the current manuscript. On the data availability section of the website, we indicated that the “Deidentified research data will be made publicly available when the study is completed and published,” not when the current protocol manuscript is accepted or published.

Upon the study's completion, de-identified data will be accessible by researchers through standard processes of the Harvard Dataverse Repository. The requesting institution’s IRB or equivalent body must approve the requested use. We think this is in compliance with PLOS One’s open data policy. Please let us know if we misunderstood your ask here.

5. We note that the original protocol that you have uploaded as a Supporting Information file contains an institutional logo. As this logo is likely copyrighted, we ask that you please remove it from this file and upload an updated version upon resubmission.

Thank you for your suggestion. We removed the logo and uploaded the file again.

6. Please review your reference list to ensure that it is complete and correct.

We have reviewed and updated the reference list.

**Reviewers:**

**Reviewer #1:** This is an interesting tool to evaluate but the overall goal of pursuing Time Limited Trials in the ED is not fully clear. The second sentence of the Discussion in the Abstract seems to be the most pertinent: "...the study intends to improve palliative care integration for seriously ill older adults in the ED and intensive care unit." The authors clearly understand the need for Goals of Care conversation and the role of Palliative Care within the ED. The introduction of time limited trials in the ED is challenging, as the clinical trajectory could be unclear early in the patient's presentation and the goals that are discussed and set forth may not align with the ICU providers' who will be responsible in carrying them out. If the main points that are to be discussed are overall patient wishes, this aligns more with the framework of a general goals of care conversation than a TLT. The authors need to delineate more specifically the exact points of discussion are to be and how these conversations are separate form a more rudimentary goals of care conversation.

We thank the reviewer for the thoughtful critique. We clarified the rationale for initiating TLT conversations in the ED in the Introduction and how we systematically refined the original TLT conversation guide to be used in this setting in the Methods:

Introduction (page 4, line 108-117):

In the ED, emergency clinicians are responsible for the initial assessment and treatment of critical conditions. Similar to other clinical initiatives that have a lasting impact on patients’ care when initiated in the ED (e.g., the first dose of antibiotics for sepsis, thrombolysis for acute ischemic stroke, etc.), for seriously ill older adults being admitted to the intensive care unit, emergency clinicians’ role is to prepare patients/surrogates for the anticipated difficult clinical course, help formulate and express their values and goals, and set the expectation to re-discuss these goals based on the clinical progression of the patient over a defined period of time. Given the limitations of the ED setting with clinical uncertainty about prognosis and anticipated outcomes, we used a comprehensive approach to refine the TLT conversation guide for use in this setting.

Methods (page 10, line 236-252):

Guided by Stirman’s framework for adapting efficacious interventions for new populations [37], we assessed the content validity of the original ICU TLT guide for use in the ED to seamlessly allow consistent care expectations and transition to the ICU team. We evaluated the following domains: necessity, clarity, relevance to the ED, and comprehensiveness. We involved three intensivists, five emergency clinicians, and three patient advisors to review and provide feedback on the refined intervention script and study approach. They rated the TLT conversation guide on a 4-point scale (e.g., 1 = irrelevant/not clear, 4 =essential/very clear) based on each section’s (1) necessity, (2) clinical relevance, and (3) clarity in the ED settings. The ratings were then used to compute a content validity index (CVI; 0–1.0, ≥0.7 is considered adequate [38]). Specifically, this expert panel identified that the following aspects are uncertain in the ED: prognosis (and sometimes diagnosis), indicators for clinical improvement, and timeframe for the TLT. Therefore, we iteratively refined these three points until they were deemed acceptable for use by emergency clinicians. As a result, the refined guide intentionally communicates the uncertainty and clinician worries simultaneously (e.g., “Based on what we know so far, our hope is that [patient] improves with these treatments. Ath the same time, I also worry the treatments may not reverse the problem.”).

**Reviewer #2:** The authors indicated, "No previous two-arm trial of TLT have been conducted; thus, the effect size remains unknown. Given the primary purpose of conducting the RCT is to demonstrate the feasibility, no gold standard for sample size calculations exists. However, prior literature suggests that 15 to 20 participants per group are required to ensure the scientific validity of the pilot study results [45].” The authors then decided to select 40 participants with 20 each intervention and control group. The authors then went on to say that “In addition, the results of this pilot study will provide information on the difference between groups and the standard deviation necessary for sample size calculation during the future full-scale study.”

These statements are contradictory. Now my question is if the study is not powered enough to see the minimum detectable effect (MID), on what basis can we use the MID estimate from a pilot study to power future studies. I agree that pilot studies have been used to estimate effect sizes to determine the sample size needed for a larger-scale randomized controlled trial (RCT) or observational study. However, this practice has been challenged because pilot study samples are usually small and unrepresentative, as this study has demonstrated by interviewing a total of 40 clinicians (20 controls and 20 interventions). There was no power analyses to arrive at those numbers which means that estimates of parameters and their standard errors may be inaccurate, resulting in misleading power calculations for even larger-scale studies. So adding that the “ the results of this pilot study will provide information on the difference between groups and the standard deviation necessary for sample size calculation during the future full-scale study.” May be problematic. Because the focus is on feasibility, results of statistical tests are generally not informative for powering the main trial outcomes. Additionally, feasibility process outcomes may be poorly estimated because the study is not powered.

The study is not powered since there was no power analysis and authors decided to only do 40 observations, so on what basis will the authors employ all these rigorous statistical analyses (two-sample t-test, binomial test of proportion, Wilcoxon rank sum test) and even run regression models. Remember the standard error estimation or precision of point estimate is based on the sample size which has effect on the estimation of p-values. So if no power analysis was done, how would you standard errors, confidence interval and p-values be reliable?

Also, note that small sample sizes can result in an imbalance between arms or within subgroups that cannot be detected with pilot data or early on in studies. Most pilot studies with smaller sample size should not be used to estimate effect sizes, provide power calculations for statistical tests or perform exploratory analyses of intervention impact.

My suggestion: I think the authors should focus on the primary objective of conducting feasibility studies which is to assess the feasibility/acceptability of Integrating the ‘Time-limited Trial’ in the Emergency Department (the time it takes to conduct the TLTs by ED clinicians) and clinician and patient-reported acceptability of the TLT but without a more rigorous power analysis, the statistical analyses at these stage should be more descriptive in personal opinion

OR if the intervention is non-evasive, you can assume MDE of say 10 percentage point or whatever based on expert advice and what you anticipate the impact of the intervention would be and go through the rigorous power analyses calculation (type I error, power of the study, design effect etc). In that case, all these inferential models can be justified

See the following references.

Teresi, J. A., Yu, X., Stewart, A. L., & Hays, R. D. (2022). Guidelines for designing and evaluating feasibility pilot studies. Medical care, 60(1), 95-103.

Kraemer HC, Mintz J, Noda A, et al. Caution regarding the use of pilot studies to guide power calculations for study proposals. Arch Gen Psychiatry 2006;63(5):484–489.

There was no specific mention of how the data will be analyzed, which is a crucial aspect that the authors should kindly include. Descriptive statistics may be examined but the specific method should be mentioned based on measurement scale of the indicator. For example, the mean and standard deviation for continuous measures (or median and interquartile range for non-normally distributed outcomes) and the frequency and percentage for categorical measures can be calculated overall and by subgroups (intervention and control) without necessarily reporting inferential statistics (p-values, etc).

We thank the reviewer for the thoughtful critique. We completely agree with the reviewer’s assessment that pilot study findings are problematic to be used for powering future efficacy trials.

As recommended by the reviewer, we clarified that our primary objectives of this pilot study are as follows:

1. To assess the feasibility of recruiting clinicians as interventionists.
2. To evaluate the feasibility of interventionists completing TLT conversations.
3. To determine the feasibility of recruiting and tracking outcomes of patients treated by the clinicians enrolled in the study.

Thus, this study's primary purpose is to serve as a feasibility assessment, ensuring that the logistical and procedural components of the larger study are viable. However, we will also assess “patient” outcomes in this pilot study. We acknowledge that the description of how we plan to measure outcomes of “patient” was insufficient. The 40 clinicians involved in this intervention are expected to take care a total of 100 patients.

In response to these considerations, the following revisions have been made to clarify these points and address the reviewer's feedback.

Methods (Page 13, line 300-321):

The current study is in Phase I of intervention development. As such, the primary purpose of this study is to demonstrate the feasibility of implementing the intervention and randomizing clinicians to become interventionists. We used the standardized framework for feasibility studies previously described [43]. The feasibility is defined as:

1. Time to complete the introduction of TLTs (primary outcome): < 15 minutes is required to complete the introduction of TLT conversation by emergency clinicians.
2. Intervention fidelity: > 75% of clinicians complete more than 75% items on TLT Fidelity checklist.
3. Clinician-reported feasibility: > 3.5 is reported by physicians on average.
4. Recruitment: > 50% of eligible and willing patients are enrolled.
5. Clinician-reported satisfaction: > 3.5 is reported by physicians on average.
6. EHR documentation by inpatient clinicians (i.e., intensivists): > 75% of the medical records of patients who received the intervention include EHR documentation related to TLT.

In addition, we seek to follow the clinical outcomes of patients under the care of our interventionist clinicians to explore the potential effect of such clinician intervention. At this stage, we needed to balance the practicality of recruiting emergency clinicians to train in the TLT conversation guide (a substantial effort for busy clinicians) and the scientific aim of demonstrating the feasibility of such a study while exploring the clinical effects (e.g., How long does it take for the interventionist clinicians to care for enough seriously ill older adults to glean at the clinical effect?). Therefore, our interdisciplinary team will recruit 20 intervention and 20 control clinicians (40 total) and follow the clinical outcomes of their patients (N=100).

We also revised this sentence:

The original sentence: (Page 16, line 344-349)

“In addition, the results of this pilot study will provide information on the difference between groups and the standard deviation necessary for sample size calculation during the future full-scale study.”

The revised sentence:

“While pilot studies cannot provide adequate power to determine the effect size for planning future studies, we intend to explore the potential clinical signal of the TLT intervention. We plan to report descriptive statistics as follows in order to estimate these as accurately as possible.”

**Table 3. Outcomes and descriptive statistics**

| **Outcomes** | **Variable Type** | **Descriptive Statistics** |
| --- | --- | --- |
| Time to complete the introduction of TLTs (primary outcome) | Continuous | Mean and Standard Deviation (if normally distributed);  Median and Interquartile Range (if not normally distributed) |
| Intervention fidelity | Categorical | Frequency (n) and Percentage (%) |
| Clinician-reported feasibility | Continuous | Mean and Standard Deviation (if normally distributed);  Median and Interquartile Range (if not normally distributed) |
| Recruitment | Categorical | Frequency (n) and Percentage (%) |
| Clinician-reported satisfaction | Continuous | Mean and Standard Deviation (if normally distributed);  Median and Interquartile Range (if not normally distributed) |
| EHR documentation by inpatient clinicians | Categorical | Frequency (n) and Percentage (%) |

Thank you again, and we look forward to hearing from you.

Sincerely,

Tadayuki Hashimoto, MD, MPH

**References**

37.	Wiltsey Stirman S, Baumann AA, Miller CJ. The FRAME: an expanded framework for reporting adaptations and modifications to evidence-based interventions. Implement Sci. 2019;14: 58.

38.	Kassam-Adams N, Marsac ML, Kohser KL, Kenardy JA, March S, Winston FK. A new method for assessing content validity in model-based creation and iteration of eHealth interventions. J Med Internet Res. 2015;17: e95.

43.	Orsmond GI, Cohn ES. The distinctive features of a feasibility study: Objectives and guiding questions: Objectives and guiding questions. OTJR (Thorofare N J). 2015;35: 169–177.
